# Supplementary material for: SUMOylation of NaV1.2 channels mediates the early response to acute hypoxia in central neurons
Source: eLife. 2016 Dec 28;5:e20054. doi: 10.7554/eLife.20054 (PMC5283832; doi:10.7554/eLife.20054)
Supplement: Supplementary file 1. — (a) Biophysical properties of INa and NaV1.2 channels cultured at 21% O2. (b) Biophysical properties of INa cultured at 7% O2. (c) Biophysical properties of INa and NaV1.2 channels expressed without the β1-subunit. DOI: http://dx.doi.org/10.7554/eLife.20054.015 [file elife-20054-supp1.docx]

**Supplementary file 1a. Biophysical properties of *I_Na_* and Na_V_1.2 channels cultured at 21% O_2_**

|  | ***I_Na_*** | | | **Na_V_1.2** | | | **Na_V_1.2-K38Q** | | | **CFP-Na_V_1.2** | | | **CFP-Na_V_1.2-K38Q** | | |
| --- | --- | --- | --- | --- | --- | --- | --- | --- | --- | --- | --- | --- | --- | --- | --- |
| **Measure** | ***I_PEAK_***  **pA/pF** | **Activation**  **V½ (mV),**  ***k*** | **SSI**  **V½ (mV),**  ***k*** | ***I_PEAK_***  **pA/pF** | **Activation**  **V½ (mV),**  ***k*** | **SSI**  **V½ (mV),**  ***k*** | ***I_PEAK_***  **pA/pF** | **Activation**  **V½ (mV),**  ***k*** | **SSI**  **V½ (mV),**  ***k*** | ***I_PEAK_***  **pA/pF** | **Activation**  **V½ (mV),**  ***k*** | **SSI**  **V½ (mV),**  ***k*** | ***I_PEAK_***  **pA/pF** | **Activation**  **V½ (mV),**  ***k*** | **SSI**  **V½ (mV),**  ***k*** |
| **O_2_ 21%** | -172 ± 20 | -23 ± 0.5,  4 ± 0.2 | -67 ± 2,  6 ± 1 | -112 ± 8 | -18.7 ± 0.2,  3.7 ± 0.1 | -59 ± 0.4,  7 ± 2 | -33 ± 7 | -4 ± 1.5,  3.6 ± 0.3 | -50 ± 1,  6.5 ± 1 | -105 ± 10 | -17 ± 2,  4.2 ± 1.8 | -61 ± 1.3,  6.6 ± 2 | -36 ± 6 | -3.4 ± 0.85,  4.2 ± 0.7 | -54 ± 3,  6.0 ± 1 |
| **O_2_ 5%** | -294 ± 25 * | -34 ± 1.5 *,  4.2 ± 0.2 | -78 ± 2*,  10 ± 2 | -192 ± 11 * | -30 ± 0.5 *,  4 ± 1 | -72 ± 1.5*,  7.5 ± 1 | -35 ± 6 * | -4 ± 2,  3 ± 1 | -49 ± 1,  6 ± 1 | -185 ± 12 | -28 ± 0.5 *,  3 ± 2 | -74 ± 3 *,  7.1 ± 1.6 | -34 ± 7 | -3.8 ± 2.5,  3.8 ± 0.7 | -51 ± 2,  5.5 ± 1.5 |
| **10 min recovery** | -290 ± 22 | -33 ± 1 *,  3.8 ± 0.2 | -75 ± 2 *,  7 ± 1 | -194 ± 14 | -32 ± 1 *,  3.9 ± 0.2 | -73 ± 1 *,  7.2 ± 2 | -36 ± 10 | -4 ± 1,  3.5 ± 1 | -51 ± 2,  6 ± 2 | -187 ± 9 | -35 ± 1.4 *,  3 ± 2 | -70 ± 2 *,  6.8 ± 3 | -32 ± 5 | -4.1 ± 1.2,  3 ± 1.5 | -50 ± 3,  5.8 ± 5 |
| **SENP1** | -42 ± 12 * | -7.5 ± 1 *,  4.1 ± 0.3 | -53 ± 1 *,  8 ± 2 | -29 ± 9 * | -2.5 ± 0.3*,  3.6 ± 0.1 | -48 ± 0.5 *,  6 ± 1 | -35 ± 7 * | -3.5 ± 1,  3.2 ± 0.4 | -49 ± 0.5,  6.5 ± 2 | -31 ± 7 | -3.2 ± 0.6 *,  3.2 ± 0.3 | -45 ± 0.9 *,  5 ± 1 | -36 ± 8 | -4.5 ± 1.5,  4.2 ± 0.8 | -50 ± 1.5,  6.6 ± 1.7 |
| **SENP1 + O_2_ 5%** | -44 ± 10 | -7 ± 1 *,  5 ± 1 | -51 ± 1.3 *,  7.8 ± 2 | -28 ± 9 | -3 ± 0.5 *,  4 ± 0.5 | -50 ± 0.8 *,  7.2 ± 1.4 | -37 ± 8 | -3.5 ± 0.8,  3 ± 0.7 | -52 ± 0.8,  8 ± 1.8 | -33 ± 5 | -4.4 ± 1.2 *,  3.5 ± 1.5 | -48 ± 1.2 *,  9 ± 2.8 | -35 ± 7 | -4.3 ± 1.4,  3.9 ± 1 | -53 ± 1.8,  6.6 ± 1.2 |
| **SUMO1** | -303 ± 17 * | -36 ± 1 *,  3.9 ± 0.2 | -77 ± 3 *,  8.5 ± 1 | -196 ± 17 * | -30 ± 0.3 *,  3.5 ± 0.2 | -69 ± 0.5 *,  7.5 ± 2 | -33 ± 5 * | -4 ± 2,  3.4 ± 0.5 | -50 ± 1.5,  6 ± 2 | -190 ± 10 | -27 ± 1.8 *,  4.2 ± 1.6 | -73± 0.8 *,  6.5 ± 4 | -34 ± 7 | -5 ± 2.4,  4 ± 1 | -52 ± 3,  6.1 ± 3 |
| **SUMO1 + O_2_ 5%** | -300 ± 18 | -37 ± 2 *,  3.7 ± 0.5 | -75 ± 2 *,  8 ± 1.5 | -193 ± 15 | -32 ± 0.8 *,  3 ± 1 | -71 ± 2 *,  8.5 ± 1 | -34 ± 6 | -3 ± 1,  5 ± 1 | -4 ± 2,  5 ± 1.5 | -186 ± 11 | -31 ± 2.1 *,  3.3 ± 1 | -70 ± 4 *,  6.4 ± 2 | -32 ± 9 | -4.8 ± 3,  3.6 ± 1.2 | -50 ± 4,  6 ± 1.8 |

**Legend**. Neurons (**Figures. 1 and 3**) or cloned channels expressed in CHO cells with the β1 subunit (**Figures. 5 and 8**) were studied in whole-cell mode. Stimulation protocols are described in the Materials and Methods. V_1/2_, the voltage evoking half-maximal conductance in mV and *k*, the slope of the curve were obtained by fitting the normalized current plotted against voltage to a Boltzmann function, I = I_max_/(1+exp [−(V−V_1/2_)/k]), where I_max_ is maximum current; and SSI, steady-state inactivation. The peak currents shown above were measured at -20 mV. Currents measured at 20 mV, a potential where the conductance was saturated under all conditions studied are shown in Table 1. The currents measured at 0 mV for CFP-Na_V_1.2 at 21% O_2_ and 5% O_2_ in pA/pF were -180 ± 11 and –178 ± 9, respectively. Data are means ± S.E.M. for 8 to 12 cells per group. *Indicates P<0.05 compared with cells studied under control conditions at ambient O_2_ for each channel type studied.

**Supplementary file 1b. Biophysical properties of *I_Na_* cultured at 7% O_2_**

|  | ***I_Na_*** | | | | |
| --- | --- | --- | --- | --- | --- |
| **Condition** | ***I_PEAK_***  **pA/pF** | **Activation** | | **SSI** | |
|  |  | **V½ (mV),**  ***k*** |  | **V½ (mV),**  ***k*** |  |
| **O_2_ 7%** | -162 ± 12 | -42.9 ± 1.5,  3.5 ± 0.5 |  | -57 ± 3,  7 ± 2 |  |
| **O_2_ 1.5%** | -267 ± 18 | -49.5 ± 1.0 *,  3.2 ± 0.8 |  | -69 ± 2,  7 ± 2 |  |
| **10 min recovery** | -262 ± 20 | -50.5 ± 1.8 *,  3.5 ± 0.5 |  | -70 ± 3 *,  6 ± 3 |  |
| **SENP1** | -44.4 ± 7 | -35.6 ± 1.0 *,  3.6 ± 0.7 |  | -51 ± 3 *,  5 ± 2 |  |
| **SENP1 +**  **O_2_ 1.5%** | -48± 8 | -34.9 ± 1.3 *,  3.5 ± 1 |  | -50 ± 4 *,  5 ± 1 |  |
| **SUMO1** | -271± 15 | -49.1 ± 0.7 *,  3.1 ± 0.8 |  | -72 ± 3 *,  5 ± 3 |  |
| **SUMO1 +**  **O_2_ 1.5%** | -273 ± 21 | -50 ± 1.3 *,  3.5 ± 0.3 |  | -72 ± 4 *  5 ± 2 |  |

**Legend**. Neurons were studied in whole-cell mode (**Figure 3**). Stimulation protocols are described in the Materials and Methods. V_1/2_, the voltage evoking half-maximal conductance in mV and *k*, the slope of the curve were obtained by fitting the normalized current plotted against voltage to a Boltzmann function, I = I_max_/(1+exp [−(V−V_1/2_)/k]), where I_max_ is maximum current; and SSI, steady-state inactivation. The peak current was measured at -20 mV. Data are means ± S.E.M. for 8 to 12 cells per group. * Indicates P<0.05 compared with cells studied under control conditions at 7% O_2_ for each channel type studied.

**Supplementary file 1c. Biophysical properties of Na_V_1.2 channels expressed without the β1 subunit**

|  | **Na_V_1.2** | | | | **Na_V_1.2-K38Q** | | | |
| --- | --- | --- | --- | --- | --- | --- | --- | --- |
|  | **Activation** | | **SSI** | | **Activation** | | **SSI** | |
|  | **V½** | ***k*** | **V½** | ***k*** | **V½** | ***k*** | **V½** | ***k*** |
| **O_2_ 21%** | -12 ± 1 | 2 ± 0.8 | -52 ± 2 | 3 ± 0.7 | -0 ± 2 | 2.3 ± 1.2 | -42 ± 3 | 3.5 ± 1.6 |
| **O_2_ 5%** | -24 ± 1.5 * | 4 ± 1 | -64 ± 1* | 3.5 ± 0.9 | -0.4 ± 1.8 | 2 ± 1 | -41 ± 2 | 4 ± 1 |
| **10 min recovery** | -26 ± 1.4 * | 4.3 ± 1.2 | -66 ± 1.5 * | 4 ± 1.8 | -1 ± 2 | 2.5 ± 1.2 | -39 ± 3 | 4 ± 1 |
| **SENP1** | -0 ± 2.2 * | 3.9 ± 1 | -40 ± 2 * | 4.1 ± 0.3 | -0.5 ± 2.5 | 2.3 ± 1.3 | -40 ± 2.55 | 3.8 ± 2.4 |
| **SENP1 + O_2_ 5%** | -0.8 ± 1.2 * | 4.1 ± 1.1 | -50 ± 2.5 * | 4.2 ± 1.7 | -2 ± 3 | 1 ± 2.7 | -37 ± 1.8 | 4 ± 2 |
| **SUMO1** | -25 ± 2.1 * | 3.2 ± 1.2 | -62 ± 1.8 * | 4.5 ± 1.6 | -1 ± 3 | 1.4 ± 2.2 | -42 ± 2.3 | 3 ± 1.5 |
| **SUMO1 + O_2_ 5%** | -27 ± 1.3 * | 3.8 ± 1 | -60 ± 2 * | 3.5 ± 1.2 | -2 ± 1 | 3 ± 2 | -39 ± 3 | 4 ± 2.3 |

**Legend**. Cloned channels were expressed in CHO cells without the β subunit and studied in whole-cell mode as described in **Figure 5**. Stimulation protocols are described in the Materials and Methods. V_1/2_, the voltage evoking half-maximal conductance; *k*, the slope of the curve were obtained by fitting the normalized current plotted against voltage to a Boltzmann function, *I* = *I_max_*/(1+exp [−(V−V_1/2_)/k]), where *I*_max_ is maximum current; and SSI, steady-state inactivation. Data are means ± S.E.M. for 5 to 8 cells per group. *Indicates P<0.05 compared with cells studied under control conditions at ambient O_2_ levels for each channel type studied.
